# Identification of a pyroptosis-related lncRNA signature in the regulation of prognosis, metabolism signals and immune infiltration in lung adenocarcinoma

**DOI:** 10.3389/fendo.2022.964362

**Published:** 2022-08-10

**Authors:** Shuyi Zhou, Yuan Cai, Zhijie Xu, Bi Peng, Qiuju Liang, Jinwu Peng, Yuanliang Yan

**Affiliations:** ^1^ Department of Hepatobiliary Surgery, Hunan Provincial People’s Hospital Xingsha Branch (People’s Hospital of Changsha County), Hunan Normal University, Changsha, China; ^2^ Department of Pathology, Xiangya Hospital, Central South University, Changsha, China; ^3^ Department of Pathology, Xiangya Changde Hospital, Changde, China; ^4^ National Clinical Research Center for Geriatric Disorders, Xiangya Hospital, Central South University, Changsha, China; ^5^ Department of Pharmacy, Xiangya Hospital, Central South University, Changsha, China

**Keywords:** lung adenocarcinoma, lncRNAs, pyroptosis, prognosis, immune infiltration

## Abstract

Pyroptosis is a cell death pathway that plays a significant role in lung adenocarcinoma (LUAD). Also, studies regarding the correlation between the expression of long non-coding RNAs (lncRNAs) and the mechanism of LUAD has aroused concern around the world. The purpose of this paper is to explore the underlying relationship of differentially expressed lncRNAs and pyroptosis-related genes. The least absolute shrinkage and selection operator (LASSO) algorithm and Cox regression were applied to construct a prognostic risk score model from the TCGA database. A pyroptosis-related five-lncRNA signature (CRNDE, HHLA3, MIR193BHG, LINC00941, LINC01843) was considered to be correlated to the prognosis and immune response of LUAD patients. In addition, the cytological experiments revealed that aberrantly expressed HHLA3 displayed a proliferation promotion role in LUAD cells A549 and H460. Next, the forest and nomogram plots have shown this lncRNA signature could be served as an independent prognostic factor for LUAD. The ROC curves further identified the prognostic value of the five-lncRNA signature. The infiltration of immune cells, such as T cells CD8, T cells CD4 memory resting, T cells CD4 memory activated and M0 macrophages were greatly different between the high-risk group and the low-risk group. It implicated that the signature is significantly effective in immunotherapy of LUAD patients. This study has supplied a novel pyroptosis-related lncRNA signature and provided a predictive model for prognosis and immune response of LUAD patients.

## Introduction

Lung adenocarcinoma (LUAD) has a high fatality rate worldwide and cannot be diagnosed at an early age. And lung adenocarcinoma is one of the common subtypes of lung cancer with increasing occurrence (1). With the development of the therapeutic approaches, including surgery, chemotherapy and radiotherapy, the 5-year survival rate of LUAD is still low, ranging from 4-17% (2, 3). Therefore, exploring the reliable approaches is very necessary for improving the diagnosis and treatment in LUAD patients.

In recent years, there is an increasing exploration in the field of pyroptosis. Pyroptosis is one of the cell death pathways that is reliable on the caspase-1 activity or caspase-4/5/11 activity (4, 5). Zhou and colleagues discovered that the cleavage of gasdermin B (GSDMB) mediated by granzyme A (GZMA) may induce pyroptosis and enhance anticancer immunity *in vitro* and *in vivo* (6). Zhang et al. has demonstrated that high expression level of GSDME could enhance the effects of cisplatin in promoting pyroptosis in A549 lung cancer cells (7). Moreover, a recent study has reported that inhibiting the expression of MELK could play a vital role in arresting the cell cycle of LUAD cells, followed by triggering the cell pyroptosis (8). Consequently, it is very necessary to explore the underlying mechanisms of pyroptosis-related gene signature in LUAD biology.

The profiles of long non-coding RNAs (lncRNAs) have been proved to participate in the biological functions of cancer cells (9). In recent years, emerging studies have investigated the underlying mechanisms and effects of lncRNAs on LUAD patients. Meng et al. reported that LINC01194 combined with miR-641could exert synergistic effects on the cell growth, migration and invasion of LUAD (10). At the same time, Tian et al. demonstrated that LINC01426 synergized with AZGP1 could exert influence on the diagnosis and prognosis of LUAD patients. The higher expression level of LINC01426 will be accompanied with poorer prognosis of LUAD patients (11). Recently, a new study has reported that lncRNAs could own the potential to be indicators of clinical cancer therapy through regulating the pyroptosis cell death pathway (12). Whereas, the roles of pyroptosis-associated lncRNAs in prognostic prediction of LUAD patients still need to be further explored.

Hence, based on pyroptosis-related 5-lncRNA signature, we constructed a potential prognostic model that could be of great value to predict the prognosis and immunological reaction in LUAD patients.

## Materials and methods

### Data collection

We searched the information from the TCGA database (https://portal.gdc.cancer.gov/repository), which included 535 LUAD tissues and 59 normal lung tissues. And the volcano plot depicted the differentially expressed genes (DEGs) between the normal and the LUAD groups (p < 0.05). We applied the “limma” package (13) to make the standardization of gene expression profiles. With the cutoff value of |fold-change| > 1 and p < 0.05, we identified the DEGs between LUAD tissues and normal lung tissues.

### Function enrichment analysis

ClusterProfiler (14) package was used to discover the biological functions. It was performed to discover the function enrichment of pyroptosis-related lncRNAs by means of GO analysis. Moreover, Xiantao tool (https://www.xiantao.love/products) was used to conduct the gene set enrichment analysis (GSEA) of the DEGs.

### Identification of pyroptosis-related lncRNAs

In order to make the identification of pyroptosis-associated lncRNAs, we firstly found 33 pyroptosis-associated genes from the previous researches (15–17). Then, the correlation between pyroptosis-associated DEGs and differentially expressed lncRNAs was explored by Pearson’s test. Pearson’s R > 0.2 was regarded as the screening criteria.

### Construction of pyroptosis-related lncRNA signature

All of 594 lncRNA-seq patients’ samples and the corresponding clinical information were searched from TCGA database. Later, 386 lncRNA-seq samples were reserved for the later analysis with precluding 149 samples with unknown statistics and 59 normal samples. 70% of the 386 samples was the training dataset and the remaining 30% was the testing dataset. Next, univariate Cox regression analysis, multivariate Cox regression analysis with least absolute shrinkage and selection operator (LASSO) were conducted to evaluate the prognostic values of pyroptosis-related lncRNAs. Then, after 10000 times boostraping, we figured out the occurrence time of each lncRNAs. Furthermore, in order to explore the prognostic value of the pyroptosis-associated lncRNA signature of LUAD patients, the analysis of multivariate cox regression was carried out on the lncRNAs that occurred above 2000 times. And the pyroptosis-related 5-lncRNA signature has been constructed. For the identification of the gene signature, R package was applied to figure out the signature containing the prognostic biomarkers. Based on the signature, the evaluation of samples’ risk score was carried out. In the training groups, risk score= blncRNA1 × lncRNA1 expression + blncRNA2 × lncRNA2 expression + · ···· + blncRNAn × lncRNAn expression (b represents the expression level of the multiplied regression model). By means of “survivalROC”, the analysis of predictive value of the pyroptosis-related lncRNA signature was depicted by receiver operating characteristic (ROC) curves at 0.5, 1, 2, 3-year survival. And, we divided the samples into two groups, the high-risk and the low-risk. Then we conducted the survival evaluation *via* the R package “survival” and “survminer”. The specificity of the pyroptosis-associated lncRNA signature combined with other clinical clinicopathological factors, such as gender, tumor stage and age could be good. Additionally, based on TCGA-LUAD database, a nomogram was built including related clinical parameters to predict the 0.5, 1, 2, 3-year survival of LUAD patients.

### Immune infiltrate analysis

The CIBERSORT (18) was applied to evaluate 22 types of tumor-infiltrating immune cells (TIICs) of the samples, including the B cells naive, B cells memory, plasma cells, T cells CD8, etc. The statistics of gene expression were downloaded from TCGA database. P < 0.05 was regarded as statistically significant. Spearman’s correlation test was used for analyzing the link between this signature’ risk score and immune cells infiltration.

### Cell culture

The LUAD cells A549 and H460 were supplied by Xiangya Cancer Center, Xiangya Hospital, Central South University. These cells were cultured in 10% fetal bovine serum and 1% penicillin/streptomycin. The cells were cultured at 37°C in a 5% CO_2_ atmosphere.

### SiRNAs for HHLA3 knockdown

The LUAD cells A549 and H460 were transfected with HHLA3 siRNAs to knock-out its expression. And the siRNA sequences were as follows: siHHLA3-1, CACGCAGGATGTGATGTCA; siHHLA3-2, GAGGATACTCAGTCAACCA.

### Cell proliferation assays

CCK-8 was applied for evaluating the cell proliferation. In brief, the 2 × 10^3^ cells were plated into the 96-well plates. Then, the cells were incubated with CCK-8 reagent for about 2 hours. Lastly, cell proliferation was evaluated using a spectrometer.

### Statistical analysis

The R Statistical Package were utilized in all statistical analysis. P < 0.05 were considered to be statistically significant.

## Results

### Identification of differentially expressed genes in lung adenocarcinoma

**Figure 1:**
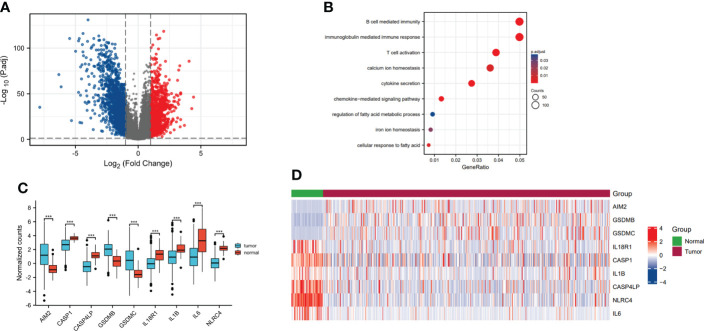
Identification of differentially expressed pyroptosis-related genes in lung adenocarcinoma. **(A)** Volcano plot showed the DEGs in TCGA-LUAD database. **(B)** Gene ontology (GO) analysis of the DEGs in TCGA-LUAD database. **(C)** The boxplot showed the expression levels of nine pyroptosis-associated genes between the normal group and tumor group. **(D)** The heatmap showed the expression profiles of pyroptosis-related genes in the normal group and tumor group. ***p<0.001.

**Figure 2:**
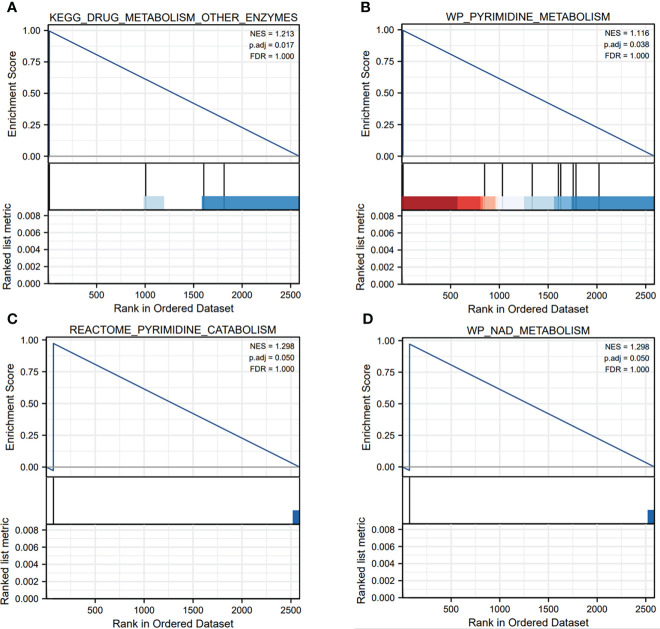
The GSEA enrichment analysis of pyroptosis-related genes in metabolism-related pathways. **(A–D)** The pyroptosis-related genes mainly participated in several metabolism-related pathways, such as drug metabolism other enzymes, pyrimidine metabolism, pyrimidine catabolism and NAD metabolism.

From the TCGA database, we identified that the 2618 genes were expressed differentially TCGA-LUAD database (**Figure 1A**, **Supplementary Table S1**). Meanwhile, we explored the biological functions *via* using the ClusterProfiler package. The GO analysis has illustrated that DEGs were mainly enriched in several immune-related pathways, including immunoglobulin mediated immune response, B cell mediated immunity, cytokine secretion, chemokine-mediated signaling pathway and T cell activation. Also, DEGs were involved in some metabolism-related pathways, such as calcium ion homeostasis, cellular response to fatty acid, iron ion homeostasis and regulation of fatty acid metabolic process (**Figure 1B**). Furthermore, the GSEA enrichment analysis portrayed that DEGs were mainly involved in two metabolism-related pathways, including drug metabolism other enzymes, pyrimidine metabolism, pyrimidine catabolism and NAD metabolism (**Figures 2A–D**).

In addition, we confirmed the differential DEGs in LUAD, such as absent in melanoma 2 (AIM2), gasdermin B (GSDMB), gasdermin C (GSDMC), etc. And we found the expression of AIM2, GSDMB and GSDMC were higher in tumor group compared to normal group, while the expression of caspase 1 (CASP1), caspase 4 like pseudogene (CASP4LP), interleukin 18 receptor 1 (IL18R1), interleukin 1 beta (IL1B), interleukin 6 (IL6) and NLR family CARD domain containing 4 (NLRC4) were lower in tumor group than normal group (**Figure 1C**). At the same time, the heatmap showed that AIM2, GSDMB and GSDMC were overexpressed in tumor group. The expression levels of IL18R1, CASP1, IL1B, CASP4LP, NLRC4 and IL6 were much higher in normal group (**Figure 1D**).

### Establishment of differentially expressed pyroptosis-related lncRNAs

**Figure 3:**
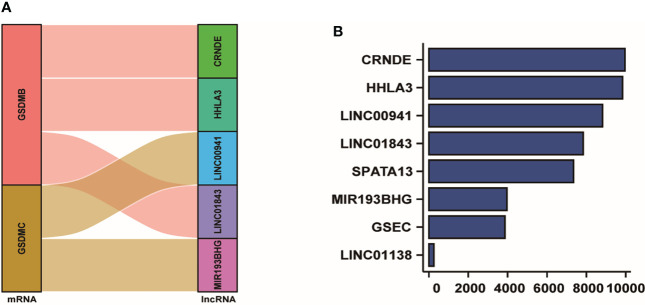
Establishment of the pyroptosis-related lncRNAs. **(A)** Sankey plot showed the association between GSDMB, GSDMC and five lncRNAs. **(B)** The frequency of all the pyrotosis-associated lncRNAs analyzed by lasso frequency analysis.

We conducted the correlation analysis of the nine pyroptosis-related genes and differently expressed lncRNAs. After conducting the Cox regression, five lncRNAs were preliminarily screened out. The Sankey plot was used to establish the relationship network between GSDMB, GSDMC and the lncRNAs. As the picture depicted, GSDMB was linked to CRNDE, HHLA3 and LINC01843. Also, GSDMC was associated with LINC00941 and MIR193BHG (**Figure 3A**). From the result of lasso frequency, we could found the frequency of CRNDE was the most, followed by HHLA3, LINC00941, LINC01843, SPATA13, MIR193BHG, GSEC and LINC01138 (**Figure 3B**). Then, we selected lncRNAs that appeared over 2000 times to construct multiple regression analysis. At last, after a multivariate Cox regression analysis, the five pyroptosis-related lncRNAs, CRNDE, HHLA3, LINC01843, LINC00941 and MIR193BHG, were picked out to establish a risk score model.

### Kaplan-Meier survival analysis of pyroptosis-related five lncRNAs

**Figure 4:**
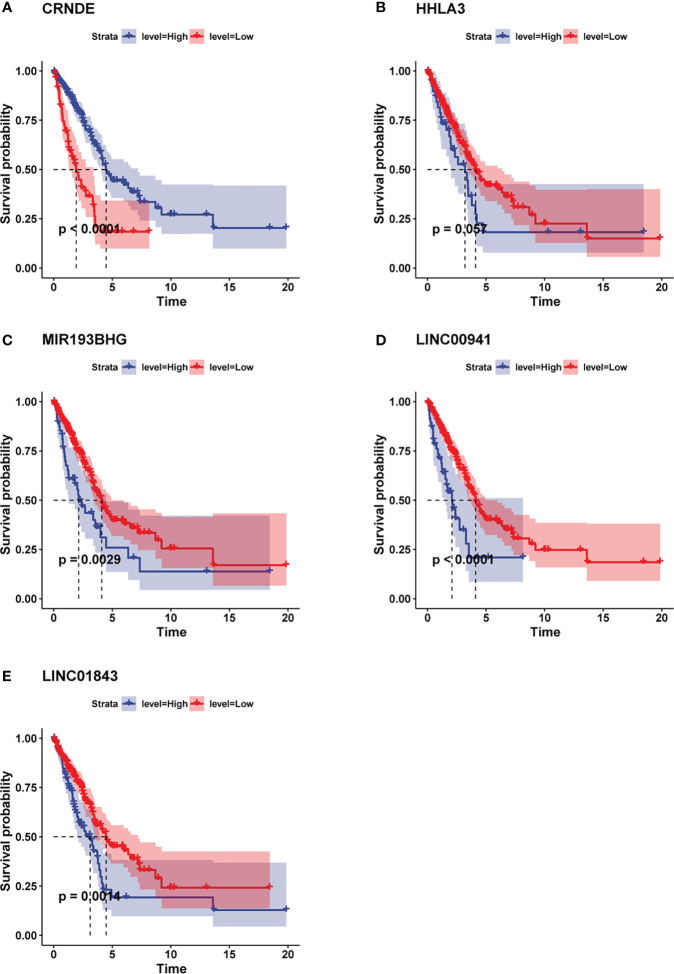
Kaplan-Meier analysis showed the prognostic values of the candidate lncRNAs. **(A)** Higher expression level of CRNDE was associated with better prognosis in LUAD patients. **(B–E)** Lower expression levels of HHLA3, MIR193BHG, LINC00941 and LINC01843 were all related to better prognosis in LUAD patients.

In order to explore whether the expression of lncRNA benefit the prognosis of LUAD patients, we applied “survminer” R package to search for the best cutoff value of each pyroptosis-related lncRNA. Then according to the cutoff value, we divided the patients into two groups: high-expression group and low-expression group, and conduct Kaplan-Meier survival analysis respectively. The findings demonstrated that the higher expression level of CRNDE was associated with better prognosis in LUAD patients (**Figures 4A**). However, the lower expression levels of HHLA3, MIR193BHG, LINC00941 and LINC01843 were all related to better prognosis in LUAD patients (**Figures 4B–E**).

### Construction of the prognostic signature based on pyroptosis-associated five-lncRNAs

**Figure 5:**
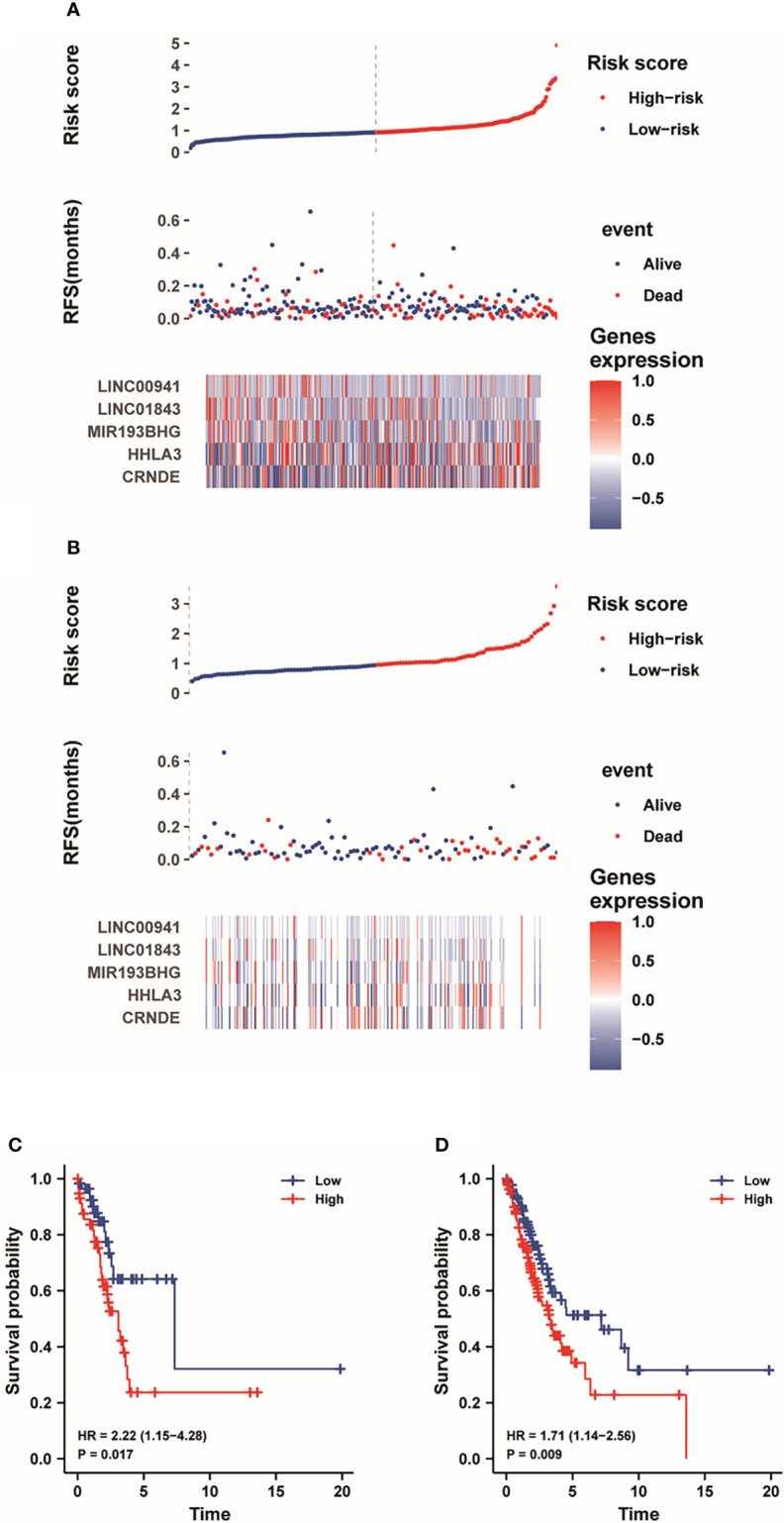
The risk score distribution and survival status of each LUAD patient. **(A**, **B)** The curve and scatter plot show the risk score of survival event of each LUAD patient in the training dataset and testing dataset, respectively. And the heatmap demonstrates the expression profiles of five lncRNAs. **(C, D)** Kaplan-Meier analysis showed the prognostic prediction of five-lncRNA signature in the training dataset and testing dataset, respectively.

Next, we used an X-tile diagram (19) to explore the cutoff value of risk score in the training group and testing group. **Figure 5A** depicted the risk score and survival event of each LUAD patient in the train group. Meanwhile, the heatmap indicated the expression profiles of LINC00941, LINC01843, MIR193BHG, HHLA3 and CRNDE in low-risk and high-risk groups. As shown in **Supplementary Figure S1**, CRNDE was downregulated while LINC00941, LINC01843, MIR193BHG and HHLA3 were upregulated in the high-risk group. In addition, **Figure 5B** depicted the risk score and survival event of each LUAD patient in the test group. At the same time, the heatmap displayed the expression profiles of five lncRNAs. Moreover, many death cases were mainly distributed in the high-risk group both in the training dataset and testing dataset (**Figures 5A, B**). Kaplan-Meier analysis depicted that the low-risk group have the higher survival probability than the high-risk group (**Figures 5C, D**). The results showed that pyroptosis-associated five-lncRNA signature could be beneficial to the prognosis of LUAD patients.

### The prognostic value of pyroptosis-associated lncRNA signature

**Figure 6:**
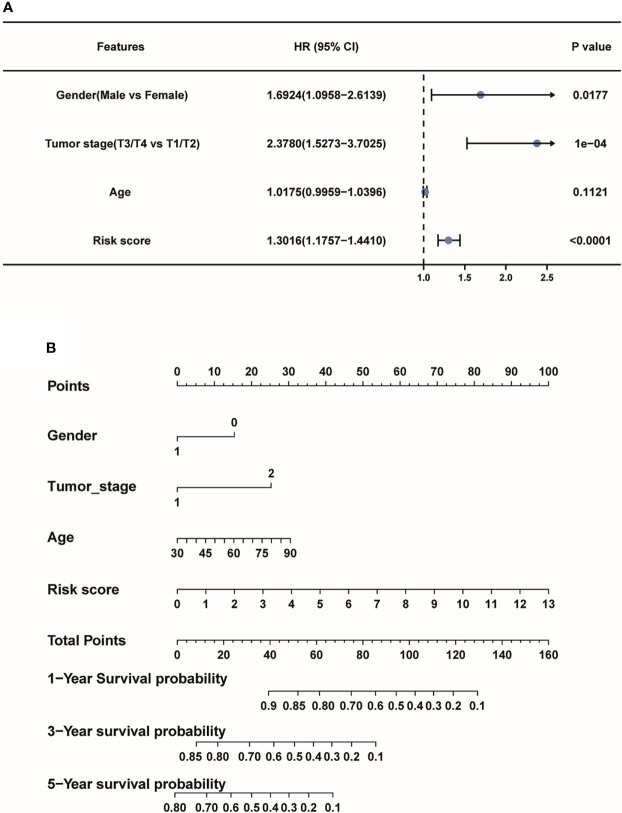
The pyroptosis-associated lncRNA signature could serve as an independent prognostic factor for LUAD patients. **(A)** The forest plot depicted the risk score model as an independent prognostic factor of LUAD patients. **(B)** The nomogram for the risk score model and other clinicopathological factors.

Then, the forest plot revealed that lncRNA-based signature (hazard ratio (HR):1.3016, 95% confidence interval (CI):1.1757-1.4410), tumor stage (HR: 2.3780, 95% CI: 1.5273- 3.7025) and gender (HR:1.6924, 95% CI: 1.0958-2.6139) were all independent factors for the prognosis of LUAD patients (**Figure 6A**). To further explore 1-, 3-, and 5-year survival probability of the lncRNA signature in the TCGA-LUAD patients, a nomogram plot was used to describe the predictive factors including the risk score model and other clinicopathological features. And the picture shows that the risk score model played the most significant role compared to other factors (**Figure 6B**). The independent findings both revealed pyroptosis-associated lncRNA signature could serve as an independent prognostic factor for LUAD patients.

**Figure 7:**
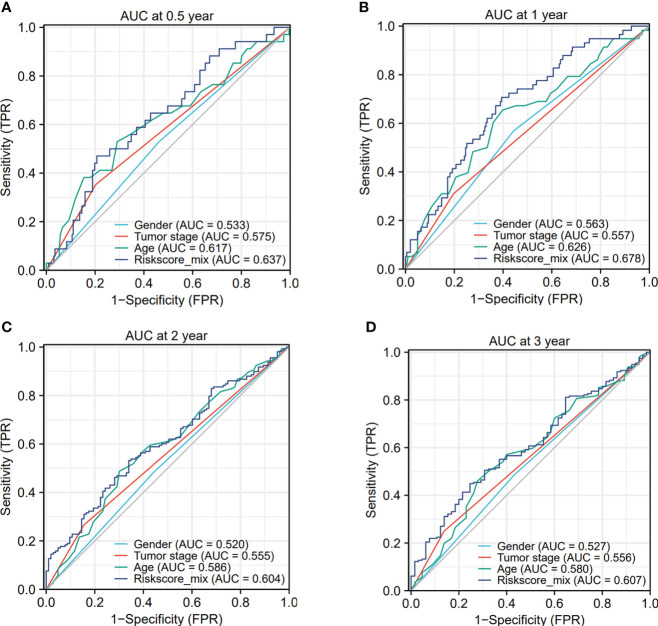
The ROC analysis depicted the area under the curve (AUC) for the gender, tumor stage, age and risk score model. **(A–D)** The AUC curves showed the prognostic prediction of risk score model and other clinicopathological factors at 0.5-, 1-, 2- and 3-year survival times.

The ROC analysis depicted that the area under the curve (AUC) for the gender, tumor stage, age and risk score model were 0.533, 0.575, 0.617 and 0.637 at 0.5-year survival (**Figure 7A**). And the AUC value of the gender, tumor stage, age and risk score model were 0.563, 0.557, 0.626 and 0.678 at 1-year survival (**Figure 7B**). The AUC value of the gender, tumor stage, age and risk score model were 0.520, 0.555, 0.586 and 0.604 at 2-year survival (**Figure 7C**). Then, the AUC of the gender, tumor stage, age and risk score model were 0.527, 0.556, 0.580 and 0.607 at 3-year survival (**Figure 7D**). Taken together, we could conclude that the sensitivity and specificity of this pyroptosis-associated lncRNA signature combined with other clinical clinicopathological factors were beneficial for clinical manage.

### Immunity analysis of the risk score

**Figure 8:**
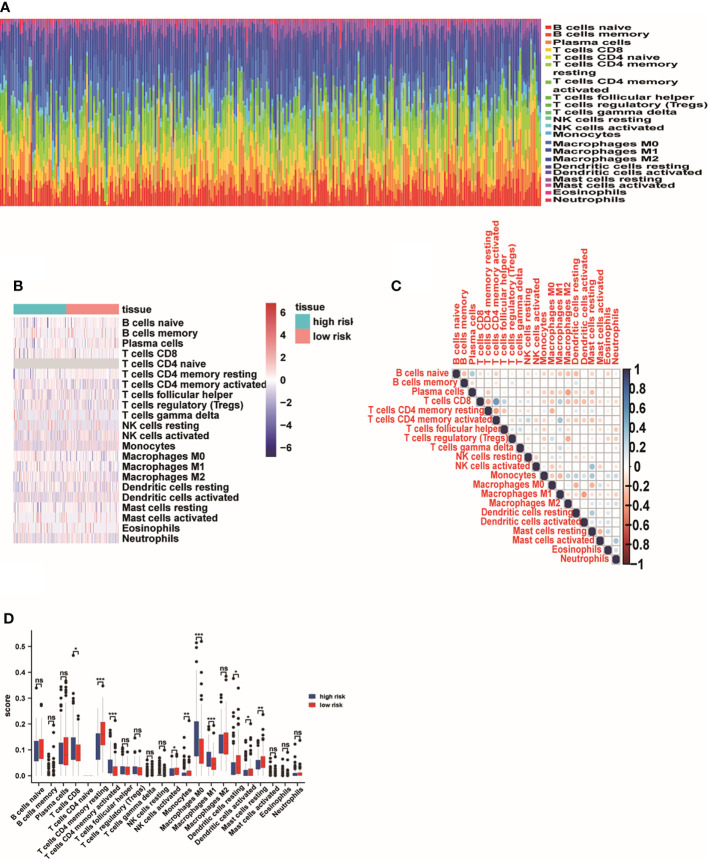
The effect of pyroptosis-associated lncRNA signature on the immune regulation of LUAD patients. **(A)** The analysis of the risk score and 22 types of TIICs in LUAD from the CIBERSORT algorithm. **(B)** The immune responses of TIICs in the high-risk group and the low-risk group. **(C)** The correlation analysis of 22 types of TIICs. **(D)** The boxplot showing the expression levels of 22 types of TIICs between the high-risk group and low-risk group. *p<0.05; ** p<0.01; ***p<0.001. ns, No Significant.

**Figure 9:**
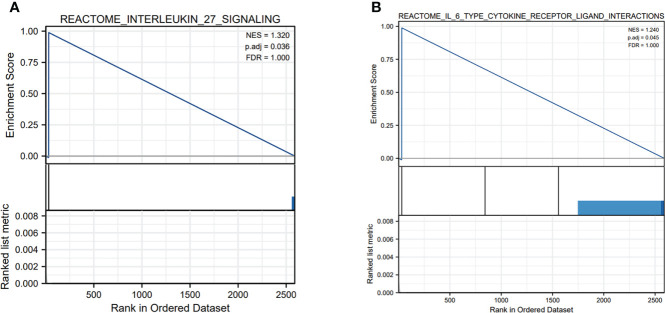
The GSEA enrichment analysis of pyroptosis-related genes in immune-related pathways. **(A, B)** The pyroptosis-related genes were mainly involved in several immune-related pathways, including interleukin (IL) 27 signaling and IL6 type cytokine receptor ligand interactions.

In order to identify the correlation of five-lncRNA signature with tumor immune responses, the evaluation of the risk score and 22 types of TIICs in LUAD from the CIBERSORT algorithm was conducted (**Figure 8A**). The heat map depicted that immune response of TIICs was quite different in the high-risk group and the low-risk group, such as T cells CD8, T cells CD4 memory resting, T cells CD4 memory activated and M0 macrophages (**Figure 8B**). From **Figure 8C**, we observed that T cells CD8 was positively related to T cells CD4 memory activated and T cells follicular helper. The findings have proven that the immune cells’ infiltration exerted great effects on the occurrence and development of LUAD patients. And the boxplot showing the scores of TIICs between the normal group and tumor group were significantly different. The expression levels of T cells CD8, T cells CD4 memory activated, M0 macrophages and M1 macrophages were higher in high-risk group compared to low-risk group (**Figure 8D**). In addition, we applied the GSEA enrichment analysis and found DEGs mainly participated in several immune-related pathways, including interleukin (IL) 27 signaling, IL6 type cytokine receptor ligand interactions (**Figures 9A, B**). Taking the findings into consideration, we concluded that the pyroptosis-associated lncRNA signature could exert effects in the immune regulation of LUAD patients.

### Knockdown of HHLA3 inhibited cell growth and increased ROS in LUAD cells

**Figure 10:**
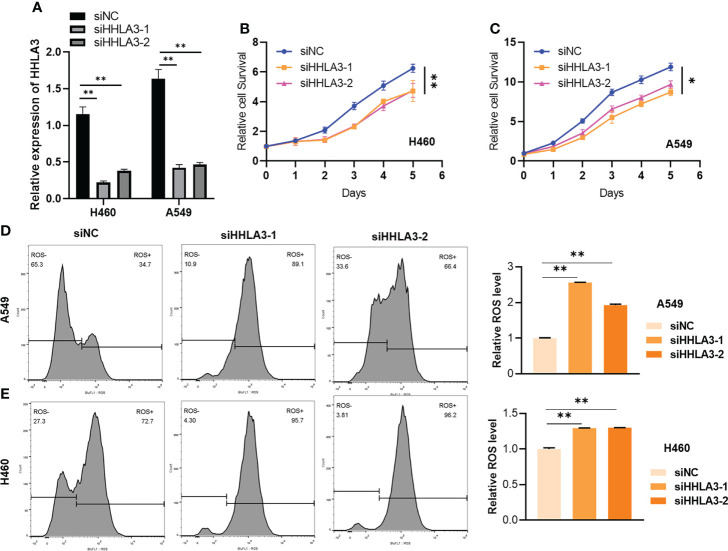
The effect of HHLA3 knockdown in A549 and H460 cells. **(A)** The A549 and H460 cells were transfected with HHLA3-targeted siRNAs. **(B, C)** HHLA3 knockdown inhibited the cell proliferation. **(D, E)** HHLA3 knockdown induced the cellular reactive oxygen species (ROS) generation. *p<0.05; ** p<0.01.

In order to evaluate the underlying biological function of HHLA3 in LUAD cell, we used siRNAs to knock-out the expression of HHLA3 (**Figure 10A**), and found that knockdown of HHLA3 significantly decrease the cell proliferation in A549 and H460 cells (**Figures 10B, C**). Moreover, the HHLA3 knockdown also increased the reactive oxygen species (ROS) generation in LUAD cells A549 and H460 (**Figures 10D, E**). The above findings revealed that HHLA3 displayed a proliferation promotion role in LUAD cells.

## Discussion

LUAD is one of the most common types of lung cancer with high mortality (20, 21). Therefore, it is high time that we should pay more attention to figure out the efficient and appropriate biomarkers regarding prognosis of LUAD patients. In this paper, we searched for a new pyroptosis-related lncRNA signature associated in LUAD cohort, which is divided into the testing group and the training group.

As is known to us, there are a few studies of lncRNA signatures in human disease that are explored and demonstrated, for example, Alzheimer’s disease and colorectal cancer (22, 23). At the same time, several lncRNA-associated signatures have been constructed in order to better predict the prognosis of LUAD patients. Jin et al. has reported that a seven-lncRNA signature (AC092794.1, AL034397.3, AC069023.1, AP000695.1, AC091057.1, HLA-DQB1-AS1, and HSPC324) could be applied to for prognostic prediction for LUAD patients (24). And Li et al. has demonstrated that an immune-associated seven-lncRNA signature (AC022784-1, NKILA, AC026355-1, AC068338-3, LINC01843, SYNPR-AS1, and AC123595-1) can be applied to predict the prognostic value of LUAD (25). Whereas, the mechanism of pyroptosis-associated lncRNAs and the LUAD prognosis has not been fully explored and clarified. This paper is to further explore the correlation between the pyroptosis-associated lncRNA signature and the LUAD patients. The findings will not only benefit the improvement of the LUAD outcomes, but also supply a newly strategy for clinical therapy. Furthermore, a few studies have demonstrated the candidate lncRNAs have strong relationship with human diseases, especially cancers. A recent study has reported that LINC00941 plays a vital role in inhibiting cell proliferation of pancreatic cancer cells (26). And the knockdown of the lncRNA CRNDE suppressed the colorectal cancer cell growth and reduced the chemoresistance by downregulating Wnt/β-catenin signaling (27).

Up to now, pyroptosis is regarded as a way of cell death that strongly linked to immune infiltration (28). 4-HBA could activate the pyroptosis pathway in A549 cells. Also, Treatment with 4-HBA could induce the caspase-1, IL1β, and IL18 encoding genes’ transcription in A549 cells (29). A newly research has implicated that in LUAD, SCGB3A2 exerts promising effects in inhibiting cell proliferation through activating the pyroptosis-associated molecular mechanism (30). In addition, pyroptosis-associated gene, GSDMD, could be served as a prognostic biomarker in LUAD (31). Pyroptosis could result in cellular contents release which was regulated by pattern recognition receptors (PRR) of tumor-infiltrating immune cells (32). A recent study has identified that the anticancer activity of GSDME could enhance the pyroptosis-related anticancer immunity *in vitro*. Pyroptosis could fight against suppression of immunity and generate systematic anticancer immunity. In this way, pyroptosis could both increase the immunogenicity of cancer cells and stimulate the immune responses (33). Pyroptosis could trigger the acute inflammation and the inflammation could enhance the immune responses (34). Another study has implicated that biomimetic nanoparticle (BNP) mediated photo-activated pyroptosis, promoting the anticancer immune responses (35). These findings could provide a novel strategy for clinical cancer immunotherapy. Therefore, exploring the mechanisms of pyroptosis in LUAD will provide a novel strategy for clinical treatment in LUAD patients.

Because of the activations of immune responses of cancer cells, immunotherapy is growing to be a great therapy for many patients with malignant tumors. Also, a large number of researches imply that the immune-associated treatment is necessary and vital for LUAD patients. Furthermore, chemotherapy and targeted therapy can be involved in regulation of tumor immune responses. Therefore, clinical trials have demonstrated that synergizing immunotherapy and other therapeutic methods seems to be a better alternative for LUAD therapy (36). In KRAS-mutant lung adenocarcinoma, the alterations of STK11/LKB1 exert effects in resisting the PD-1/PD-L1 inhibitors. It points out that the combination of immunotherapy will be used in the subset of LUAD group (37). Moreover, various studies have shown that tumor-infiltrating cells are significant in activating immune changes of tumor microenvironment, such as T cell alterations and lung cancer-related macrophages (38). A large number of “pre-exhausted” to exhausted T cells are linked to better LUAD prognosis. Whereas, tumor regulatory T cells including IL1R2 are correlated to poorer LUAD prognosis (39). And the deficiency of B cells and the accumulating of M0 macrophages have a relationship with poor prognosis of LUAD (40). This study found that high expression levels of T cells and M0 macrophages were found in the high-risk group, indicating immune tolerance in the high-risk LUAD patients. Therefore, the pyroptosis-associated lncRNA signature could provide a novel promising strategy for clinical anticancer immunotherapy. Whereas, there still needs further verification to explore the effects of the signature in prognostic prediction of LUAD patients.

However, our study still has a few limitations. It is important to explore the specificity and accuracy of this pyroptosis-related lncRNA signature for immune infiltration and prognosis of LUAD patients. At the same time, the guiding principle about the risk score model in clinical application should be further verified.

## Conclusion

To sum up, a new pyroptosis-related five-lncRNA signature could be established for prognostic prediction of LUAD patients as a promising technical strategy. It is strongly correlated to risk scores and other clinical characteristics. And the relationship between the signature value and immune-infiltration cells has been clarified by immune studies. In this study, the following statistics have proven the correlation between pyroptosis-related lncRNAs and LUAD patients’ prognosis and immune responses. It will supply a novel prediction approach for the LUAD treatment.

## Data availability statement

The original contributions presented in the study are included in the article/**Supplementary Material**. Further inquiries can be directed to the corresponding authors.

## Author contributions

SZ, YC, ZX, BP and QL: Conceptualization, Data curation, Methodology, Writing-Original draft preparation. SY and YC: Visualization, Investigation. ZX: Supervision, Resources. YY: Formal analysis, Funding acquisition. YY and JP: Software, Validation. YY and JP: Writing- Reviewing and Editing.

## Funding

This study is supported by grants from the Natural Science Foundation of Hunan Province (2021JJ30904) and the horizontal project (2021-021, 1 43010100).

## Conflict of interest

The authors declare that the research was conducted in the absence of any commercial or financial relationships that could be construed as a potential conflict of interest.

## Publisher’s note

All claims expressed in this article are solely those of the authors and do not necessarily represent those of their affiliated organizations, or those of the publisher, the editors and the reviewers. Any product that may be evaluated in this article, or claim that may be made by its manufacturer, is not guaranteed or endorsed by the publisher.

## Supplementary material

Supplementary Figure 1 The boxplot showed the expression levels of 5 lncRNAs between the high-risk group and low-risk group.
