# Supplementary figures and images for: Identification of a pyroptosis-related lncRNA signature in the regulation of prognosis, metabolism signals and immune infiltration in lung adenocarcinoma
Source: Front Endocrinol (Lausanne). 2022 Aug 10;13:964362. doi: 10.3389/fendo.2022.964362 (PMC9401518; doi:10.3389/fendo.2022.964362)

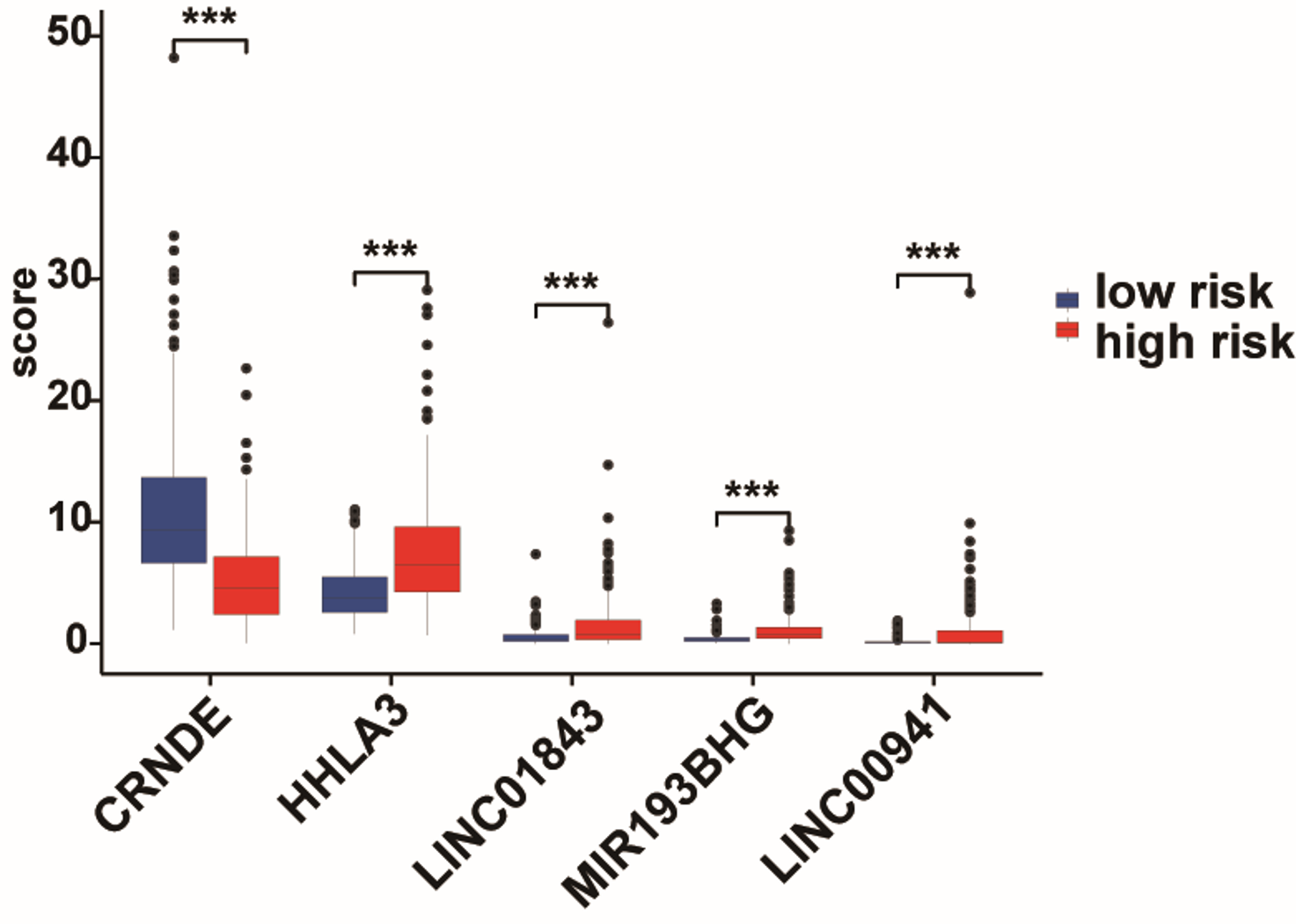

Supplement: Supplementary Figure 1 — The boxplot showed the expression levels of 5 lncRNAs between the high-risk group and low-risk group. [file Image_1.tif]
